# Mesenchymal stem cells as potential therapeutic approaches in celiac disease

**Published:** 2016-12

**Authors:** Ali Moheb-Alian, Flora Forouzesh, Mohammad Rostami-Nejad, Kamran Rostami

**Affiliations:** 1 *Basic and Molecular Epidemiology of Gastrointestinal Disorders Research Center, Research Institute for Gastroenterologyand Liver Diseases, Shahid Beheshti University of Medical Sciences, Tehran, Iran*; 2 *Department of Genetics, Tehran Medical Sciences Branch, Islamic Azad University, Tehran, Iran*; 3 *Gastroenterology and Liver Diseases Research Center, Research Institute for Gastroenterology and Liver Diseases, Shahid Beheshti University of Medical Sciences, Tehran, Iran*; 4 *Department of Gastroenterology Milton Keynes University Hospital, United Kingdom*

**Keywords:** Celiac disease, Cell therapy, Immunological pathways

## Abstract

As a chronic immune complication, celiac disease has a broad spectrum of clinical manifestations and gluten ingestion as an external trigger will induce the onset of this disease in genetically predisposed individuals. Because of the complex nature of celiac disease and various cascades of immunological pathways, therapies which are tend to target a single pathway or factor, often have unsatisfactory results. Thus, it should be considered that the new emerging area of cellular therapy by targeting multiple pathways may hold the key for treating celiac affected patients with complicated forms of this disease. The aim of this review is to discuss different pathways which are affected by celiac disease and to compare how various strategies, mainly cellular therapies, can regulate these pathways.

## Introduction

Celiac disease is a prevalent and immune mediated intestinal disorder with complicated genetic backgrounds (1). The onset of disease is induced by ingestion of gluten, which is mainly found in rye and barley (2). Celiac disease has a wide spectrum of clinical manifestations which can vary from asymptomatic to severely symptomatic classical form of celiac disease. Its diagnosis is based on serological tests for anti-tissue-transglutaminase (tTG) antibodies and anti-endomysial (EMA) (3) antibodies and is confirmed by the endoscopy and biopsy of small intestine. In celiac disease HLA-DQ molecules bind to gluten derived peptides and present them to antigen-specific T cells(4), then the inflammatory responses arising from HLA-DQ and gluten complex consist of lymphocytic infiltration of the lamia propria, increase in intraepithelial lymphocyte population, hyperplasia of crypts and flattening of villi (5) which is caused by the destruction of enterocytes (6). Refractory state of CD develops in small percentage of adult patients (2- 5%) and despite strict adherence to GFD there is a significant raise in IELs which can develop to enteropathy associated T-cell lymphoma (EATL) (7). There are 2 identified group of refractory CD patients (8): Refractory CD 1 are those with normal IEL and the refractory CD 2 are those with lacking expression of surface CD8 and CD3 which can be regarded as cryptic lymphoma. CD treatment is mainly based on a gluten free diet (GFD) which is troublesome for affected patients because of the lifelong interventional regimen (9). Due to the fact that the complex cascades of immunological pathways which are responsible for the destruction of enterocytes, the newly developed biological and chemical therapies often have unsatisfactory effects, mainly because they tend to target a single pathway instead of the modification of the multiple pathways. The aim of this study is to discuss different pathways which are affected by celiac disease and to compare how various strategies, mainly cellular therapies can regulate those pathways.

**Intestinal Regeneration**

**Figure 1:**
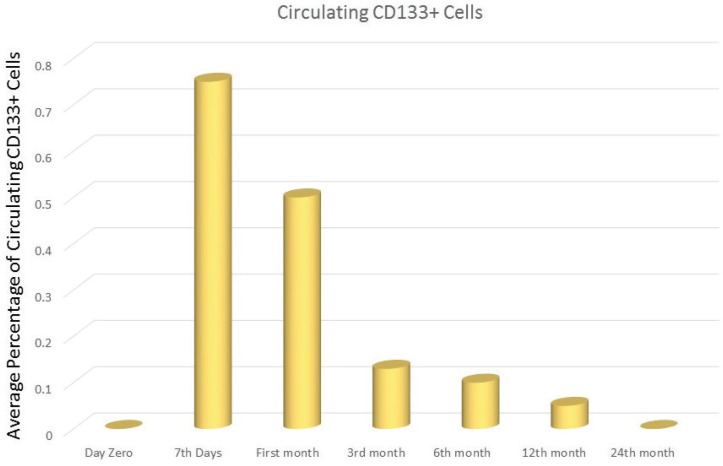
Longitudinal changes of circulating CD133 during 24 months’ GFD in CD patients

**Figure 2:**
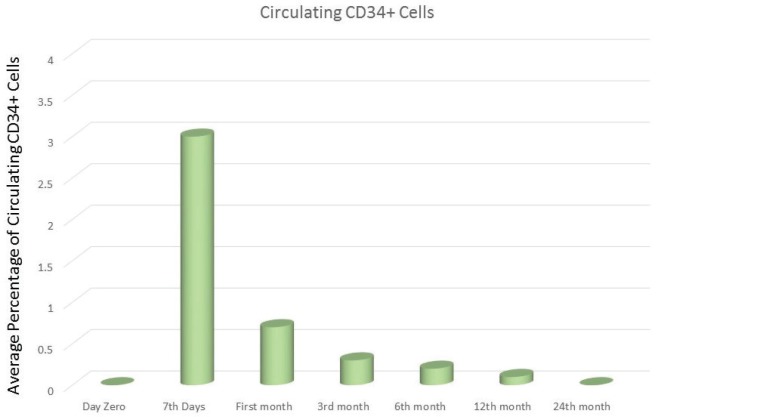
Longitudinal changes of circulating CD34 during 24 months’ GFD in CD patients

The emerging area of cellular therapy for CD is mainly base on the stem cell therapy which has the advantage of targeting multiple pathway and has yielded the promising results. It is crucial to bear in mind that the intestinal tract has a highly regulated process for the regeneration mainly due to the harsh environment which it is exposed. All the differentiated epithelial cells of the intestine derived from a single intestinal stem cell (ISC) (CD133+/Lgr5+ crypt cell) (10) compartment which resides at the crypt base. The amplifying cells that are generated from ISC migrate upward, increasingly lose their proliferative capability and become differentiated villous epithelial cells. Studies show that a normal putative ISC density is about 0.5-1 CD133+ or Lgr5+ cell per crypt and less than 0.5 CD133+ or Lgr5+ cell per crypt in active celiac patients and upon starting the GFD the number of CD133+ and Lgr5+ significantly increased at 6 months and reached its peak at 12 months of diet (11). It is also mentioned that the traffic of circulating CD34+ hematopoietic stem cell (HSC) increased in active CD patients comparing to the healthy control group (11). This increase may be related to prevalence of apoptotic versus survival programs which HSC represents a supplementary ISC source upon depletion of CD 133+/Lgr5+ crypt epithelial cells in patients with active CD(12). Interestingly, the significant increase of circulating HSC in the first week of GFD is suggesting that bone marrow derived stem cells play a major role at the initiation of the enteric repair when the ISC source is depleted, afterward the circulating HSC traffic is progressively decreased due to expanding of the local ISC compartment. Thus it can be concluded that bone marrow originated stem cells represent a potential source for intestinal regeneration (figures 1 and 2) (13).

Stem cell transplantation is an effective treatment for patients with severe refractory autoimmune diseases compare to conventional treatments like ineffective GFD regimen for patients with refractory CD and enteropathy- associated T cell lymphoma (14). Considering the ethical complications using the embryonic stem cells, mesenchymal stem cell (CD34-) and hematopoietic stem cell (CD34+) are recommended as the best candidate for clinical application.

**Hematopoietic Stem Cell**

HSCs are a heterogeneous population of cell that are derived from mesoderm and can produce both the myeloid and lymphoid lineages of blood cells. HSCs are shown to play a prominent role in mucosal healing (15) due to their ability to produce different cell population including epithelial cells, vascular cells and pericryptal myofibroblasts (16). In active CD the increased number of peripheral traffic of CD34+ HSCs did not correlate with the level of anti-tTG and severity of histological damage and it’s mainly due to the increase cell death in the mucosa (17). Although it is conceivable that HSC transplantation induces immune tolerance, it’s crucial to consider clearing body from committed lymphocyte clones beforehand, thus after immune reconstitution the pathogenic clones will never reappear. In the context of clinical application HSC transplantation has been examined on few CD patients with complicated forms of disease like EATL and refractory. In patients with refractory CD, interleukin (IL) 15 has shown to play an important role in promoting T cell cytotoxicity and is overexpressed in celiac mucosa (18). The overexpression of IL 15 leads to resistance of effector T cells to the CD4+ CD25+ transcription factor (FOXP3+) which inhibits the regulatory T cells from their suppressive activity. It seems that IL15 by activation of JAK3 and STAT5 (19), facilitated the emergence of aberrant IEL population. Also IL15 induces the expression of anti-apoptotic BCL which in turn inhibits IEL from apoptosis and leads to their malignant progression (20). HSC transplantation by rescuing patients from developing an overt lymphoma seems beneficial for patients with type II refractory CD but has proven unsatisfactory for patients with EATL (21). Studies on allogenic HSC transplantation have proved that after transplantation, normalization of both cytokines profile and FOXP3+ T cell is observed and gliadin stimulation did not induce proliferation of T cells. These data suggest that allogenic HSC transplantation can lead to induction of immune tolerance to oral antigens. HSC transplantation is an intensive treatment which is aimed not only at regeneration of gut mucosa but at resetting the immune system. Follow up studies showed the occurrence of relapses, mainly in autologous setting and high potential risk of mortality. These limitations make this treatment unsuitable for non-life threatening condition as the first line of therapy (21).

**Mesenchymal Stem Cell**

Mesenchymal stem cells (MSCs) are multi-potent stromal cells that can differentiate into a variety of cell types like myocyte, adipocyte, osteoblast and chondrocyte (22). They can be isolated from adipose tissues, fetal tissues, muscle connective tissue, placenta and umbilical cord (23). Three minimal criteria need to be met for a cell to be identified as MSC: MSCs need to be plastic-adherent under standard culture condition, MSCs have to differentiate to adipocytes, osteoblasts and chondroblasts in vitro (24), MSCs should express CD105, CD73, CD90 and lack surface expression of CD45, CD34, CD14, CD11b, CD 19 and HLA-DR. The lack of immunogenicity makes MSCs more promising than HSCs and their transplantation can be achieved in the absence of myeloablative conditioning (25). It was shown that they have strong modulatory effects on all immune cells together with a potential regenerative effect, which make them a more suitable option for transplantation (26). Due to their lack of expression of MHC class 2 and minimal expression of MHC class 1 antigens and absence of the expression of costimulatory molecules like CD40, CD80 and CD 86, MSCs have the ability to cross the HLA barriers for transplantation and due to their anti-inflammatory and modulatory activity, they can create a microenvironment called “quansiniche“ to prime naïve immune cells toward tolerogenic profile (27). The protective effects of MSCs are via the paracrine exertion of protective molecules like indoleamine 2,3 dioxygenase (IDO), prostaglandin E2 (PGE2), nitric oxide (NO) and insulin like growth factor rather than differentiating them to end-organ cells(28). Furthermore, expressing the HLA-G (29) molecule are able MSCs to induce apoptosis by CD8+ T cells and inhibit their proliferation and suppressing the NK cell lytic activity, dendritic cell maturation (30) and inducing the expansion of T regulatory cells (31). Celiac disease is a chronic disease which both innate and adaptive immune responses are engaged thus it is crucial to discuss different scenarios that MSCs can exert their protective effects. The studies recommended that the inhibitory function of MSCs on IL15 secretion and function will protect the patients with refractory CD from developing EATL (31).

**The Epithelial Barrier**

Maintaining the selective permeability is achieved by the complex interplay among epithelial cells in intestine. Epithelial barrier is consisting of both tight and adherent junctions that connecting the adjacent enterocytes. Pro- inflammatory cytokines can disrupt both complexes via phosphorylation and in turn increase the permeability of intestine due the opened tight junctions. Studies have shown that in mouse model of colitis, MSCs can exert their protective effect by reassembling claudins which have the most prominent role in tight junctions and thus maintaining the epithelial barrier (32). Moreover, the secretions of IL6, hepatocyte growth factor (HGF) and vascular endothelial growth factor (VEGF) by MSCs can inhibit the interaction of the FAS receptor with its ligand thus avoid the activation of caspase-3 and caspase-8 which can leads to enterocyte apoptosis that is responsible for villous atrophy in CD (33).

**Natural Killers and Intraepithelial Lymphocytes**

The intraepithelial lymphocytes are normally found sparsely across small intestinal mucosa and have a crucial role in innate immune response. Their increasingly activation in CD leads to epithelial damage. Through production of IFN-y, perforin and granzymes, CD8+ TCRaB+ cells induce apoptosis in enterocytes (34). Also the released IL15 from enterocyte upon gluten stimulus, upregulates the expression of the key receptors involved in IEL-mediated destruction of enterocytes which are the natural killer (NK) receptors NKG2D (NK cell receptor D), and CD94–NKG2A (natural killer cell antigen CD94– NK cell receptor A)(35). The stress signals expressed by the enterocyte involved MHC class I-related chains (MIC) A and B, and HLA-E, which act as the natural killer receptors ligands (36). As the result enterocytes become the target for “dual cytolytic effect” by IELs. By downregulating the expression of NKp44, NKp30 and NKpG2D receptors and moreover by suppressing the production of IFN-Y, MSCs possess the ability to inhibit the natural killer cells cytotoxicity (36). Although it has proven that MSCs are susceptible to lysis by IL2 activated natural killers but this lysis is inhibited by excessive amount of IFN-Y found in celiac mucosa, thus suggesting the favorable microenvironment for MSCs (37).

**Antigen-Presenting Cells**

HLA genes are the most powerful susceptibility determinants for CD predisposition (38). HLA-DQ2 and HLA-DQ8 are the main predisposing genes for developing celiac disease which if expressed in heterozygous setting can stimulate broader T-cell Repertoire (39). The expression of these HLA molecules on dendritic cells with high affinity for deamidated gluten derived peptides facilitate their presentation to CD4+ T cells. MSCs can impair monocyte differentiation into dendritic cells via either blocking the G0 to G1 cell cycle or by exerting the inhibitory soluble proteins involving IL6, PGE2 and CSF (40). Moreover, these soluble factors can shift mature dendritic cell toward less mature phenotypes with less expression of CD40, CD80, CD83, CD86 and HLA class 2 molecules on their surface. Thus this shift toward more tolerogenic profile can potentially avoid T cell activation and subduing inflammation in CD (41).

**B cell-lymphocyte**

Intestinal plasma cells produce the IgA specific for tissue transglutaminase and gluten derived peptides which is the hallmark for active CD. MSCs derived chemokine involving CCL2 and CCL7 inhibit STAT 3 and thus can suppress B cells proliferation and differentiation into plasma cells (42). Moreover, by blocking B cells in G0 to G1 cycle, MSCs are interfering with immunoglobulin production. MSCs inhibitory effects on B cells are also due to the activity of IDO that is exerted from MSCs that in turn interferes with B cell proliferation by depriving them from tryptophan (43).

**Figure 3:**
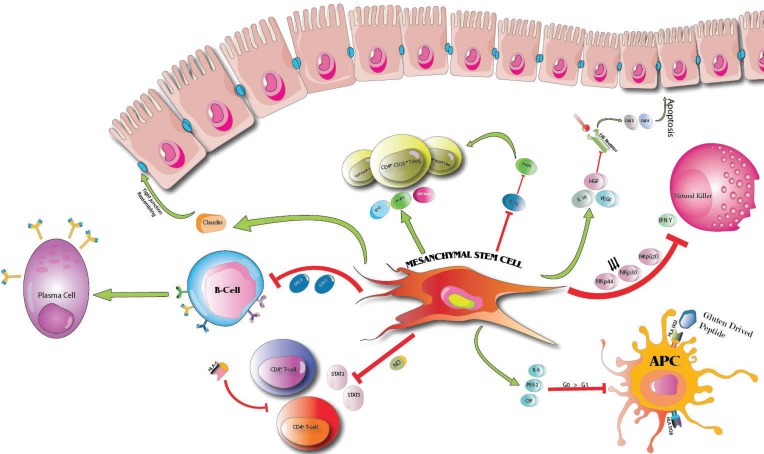
MSCs modulation of immune response. Modulation of immune system response through intraction of MSCs with all of the immune cells involved in celiac pathogenesis, consisting of B-cells, regulatory T-cells, T lymphocytes and endothelium. Inhibitory effects of MSCs depend on cell to cell intraction and via different factor and chemokines like: NO, CCL2 and CCL7, FoxP3, HLA G, IFN Y, IL16 and IL6, IDO, CSF and PEG2. Also via claudin for reassembling tight junctions

**Figure 4:**
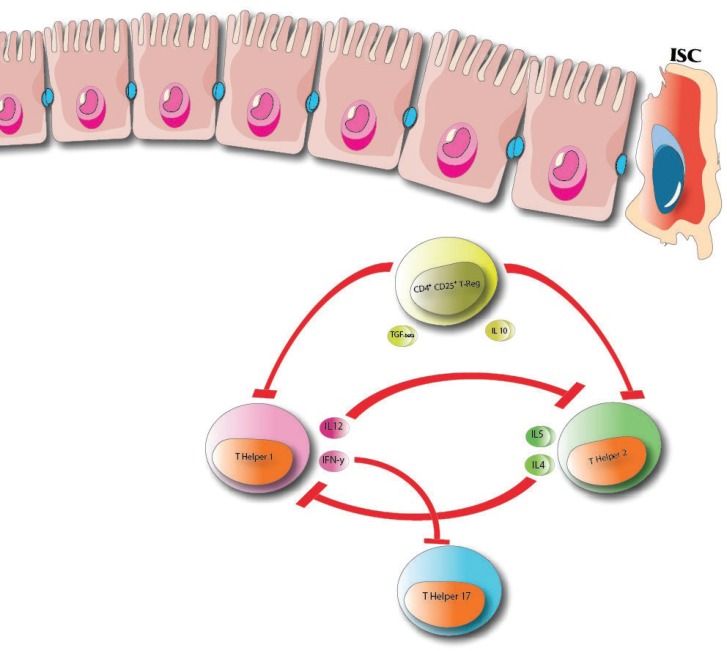
Immunomodulation of T cell response. TGF-beta and IL 10 exerted from regulator T cells inhibit promotion of T-helper 2 and T-helper

1. IL 12 and IFN-Y exerted from T helper 1 inhibits promotion of T-helper2 and T-helper 17 respectively. IL 4 exerted from T-helper 2 inhibits the promotion of T-helper 1. MSCs induce its beneficial effect through immunomodulation of T cell response by shifting the Th1/Th2 ratio toward Th2 profile.

**T Reg (Regulatory T cells)**

In vitro studies have shown that MSCs can express various extension of FoxP3 depending on culture condition that in turn increases T regulatory population(44). Furthermore, MSCs also share the ability to secret TGF-B with regulatory T cells with the same modulating effects (45).

**T Cell Response**

MSCs induce its beneficial effect through immunomodulation of T cell response by shifting the Th1/ Th2 ratio toward Th2 profile (46). IFN-y exerted from Th1 can suppress Th17 differentiation as one the pro- inflammatory cytokine and also by suppressing the IL 12 which suppresses Th2 differentiation, MSCs shifts the Th1/ Th2 ratio toward Th2 profile (46). Also effects of MSCs on T cells are mainly due to the activity of IDO which inhibits T cell differentiation via deprivation of tryptophan and the PGE2, exerted from MSCs, shifts Th1 toward IL4 secreting profile to suppress Th1 differentiation (47). Also TNF-α down regulation, due to the presence of MSCs which is overexpressed in refractory CD mucosa, can help CD patients from developing severe complicated form of celiac disease (figure 3 and 4) (48).

## Conclusion

As it has been demonstrated in this review, by possessing a wide range of immunomodulation properties, MSCs can target almost all the mechanisms involved in CD pathogenesis. Although it needs to be considered that MSCs apply their actions in a specific mucosal microenvironment. Due to their cell to cell interaction and by releasing a wide range of immunoregulatory substances, it’s not essential for MSCs to be persistence in the damaged mucosa. It is worth mentioning that the most common rout of MSCs delivery in their therapy is via intravenous injection and in many occasions it has been shown that MSCs were trapped in the lungs due to their big size. Furthermore, studies have shown no biological differences regarding the sources which MSCs are obtained, but MSCs transplantation is an invasive procedure and there are many potential limitations when they are obtained from bone marrow and adipose tissue. These limitations have given rise to the need of obtaining MSCs from other sources with unlimited donors, including umbilical cord blood, amniotic fluid, amnion and placenta which has the best proliferative properties. Finally, further methodological variables such as the rout, doses and intervals of administration need to be tuned for the best approach before therapeutic prospect of using MSCs as the clinical therapy for celiac disease.
